# Anesthetic Agents and Cardiovascular Outcomes of Noncardiac Surgery after Coronary Stent Insertion

**DOI:** 10.3390/jcm9020429

**Published:** 2020-02-05

**Authors:** Hyun-Kyu Yoon, Kwanghoon Jun, Sun-Kyung Park, Sang-Hwan Ji, Young-Eun Jang, Seokha Yoo, Jin-Tae Kim, Won Ho Kim

**Affiliations:** Department of Anesthesiology and Pain Medicine, Seoul National University Hospital, Seoul National University College of Medicine, Seoul 03080, Korea; hyunkyu18@gmail.com (H.-K.Y.); tonyjj88@gmail.com (K.J.); mayskpark@gmail.com (S.-K.P.); taepoongshin@gmail.com (S.-H.J.); na0ag2@hotmail.com (Y.-E.J.); muroki22@gmail.com (S.Y.); jintae73@gmail.com (J.-T.K.)

**Keywords:** surgery, anesthesia, coronary stent, major adverse cardiovascular event

## Abstract

Patients undergoing noncardiac surgery after coronary stent implantation are at an increased risk of thrombotic complications. Volatile anesthetics are reported to have organ-protective effects against ischemic injury. Propofol has an anti-inflammatory action that can mitigate ischemia-reperfusion injury. However, the association between anesthetic agents and the risk of major adverse cardiovascular and cerebral event (MACCE) has never been studied before. In the present study, a total of 1630 cases were reviewed. Four different propensity score matchings were performed to minimize selection bias (propofol-based total intravenous anesthesia (TIVA) vs. volatile anesthetics; TIVA vs. sevoflurane; TIVA vs. desflurane; and sevoflurane vs. desflurane). The incidence of MACCE in these four propensity score-matched cohorts was compared. As a sensitivity analysis, a multivariable logistic regression analysis was performed to identify independent predictors for MACCE during the postoperative 30 days both in total and matched cohorts (TIVA vs. volatile agent). MACCE occurred in 6.0% of the patients. Before matching, there was a significant difference in the incidence of MACCE between TIVA and sevoflurane groups (TIVA 5.1% vs. sevoflurane 8.2%, *p* = 0.006). After matching, there was no significant difference in the incidence of MACCE between the groups of any pairs (TIVA 6.5% vs. sevoflurane 7.7%; *p* = 0.507). The multivariable logistic regression analysis revealed no significant association of the volatile agent with MACCE (odds ratio 1.48, 95% confidence interval 0.92–2.37, *p* = 0.104). In conclusion, the choice of anesthetic agent for noncardiac surgery did not significantly affect the development of MACCE in patients with previous coronary stent implantation. However, further randomized trials are needed to confirm our results.

## 1. Introduction 

Patients who undergo noncardiac surgery after coronary stent insertion are at an increased risk of perioperative cardiovascular complications [1,2]. The risk is aggravated by the increased incidence of stent thrombosis or excessive bleeding due to stopping or maintaining antiplatelet agents [3,4,5]. A recent guideline specifies the optimal timing of elective noncardiac surgery after coronary stent implantation and optimal perioperative regimens for dual antiplatelet therapy [3]. The rate of occurrence of perioperative major adverse cardiovascular and cerebral event (MACCE) after noncardiac surgery has decreased over the years [6]. However, the incidence of MACCE after noncardiac surgery in patients with previous coronary stent implantation is still as high as 6.1%–15% [4,5] and the efforts to identify the patients who are at risk of perioperative complications and reduce their mortality rate [7,8,9,10] remain ongoing. 

Volatile anesthetics have shown organ-protective effects, including that of the heart [11,12,13]. Propofol has anti-oxidant and anti-inflammatory actions; therefore, it has the potential to attenuate the ischemia-reperfusion injury of major organs [14,15,16]. Since cardiovascular complication after surgery involves sympathetic activation, hypercoagulability, arterial thrombosis, and inflammation [17,18,19], the choice of anesthetics has potential to affect the incidence of MACCE after noncardiac surgery in patients with a history of coronary stent insertion. Previous animal studies have demonstrated that volatile anesthetics could reduce the size of myocardial infarction [11,12]; however, clinical studies have reported conflicting results about their effects in patients undergoing noncardiac surgery [20,21,22]. A recent large international randomized trial revealed that volatile anesthetics in cardiac surgical patients did not provide significant cardioprotection compared with total intravenous anesthesia (TIVA) [23]. However, to our knowledge, there have been no studies comparing the protective effects of TIVA and volatile anesthetics in patients undergoing noncardiac surgery after coronary stent implantation. Therefore, it would be reasonable to investigate the association between different general anesthetic agents used during the surgery and respective clinical outcomes in such patients. 

For this purpose, we performed a retrospective cohort study to investigate the relationship between the choice of general anesthetics, including TIVA, sevoflurane, and desflurane used for noncardiac surgery and the incidence of MACCE after noncardiac surgery in patients with a history of coronary stent implantation. 

## 2. Materials and Methods

### 2.1. Study Design

The institutional review board (IRB) of Seoul National University Hospital approved this single-center retrospective observational study (1907-076-1047). Written informed consent was waived by the IRB due to the retrospective study design. The electronic medical records of the patients who underwent noncardiac surgery between 2004 and 2016 within a period of five years after coronary stent implantation were reviewed. Coronary stent types included bare-metal and drug-eluting stent of first- and second-generation. Patients were excluded if the operation time was less than one hour to exclude the cases with little anesthetic exposure or if the information regarding the type of stent or time of stent implantation was not available. Patients who were administered other antiplatelet agents preoperatively, in addition to aspirin and clopidogrel, were also excluded from our study (cilostazol *n* = 69, sarpogrelate *n* = 6). This was done to evaluate the association between maintenance/discontinuation of aspirin/clopidogrel and MACCE without the effect of other antiplatelet agents. Another reason was that the number of patients dosed with other antiplatelet agents was small. There were no patients who were administered ticagrelor or prasugrel. 

### 2.2. Data Collection

Patient demographics and data on potential as well as previously established risk factors for MACCE were extracted from the electronic medical records (Table 1) [1,24], including time interval between percutaneous coronary intervention and surgery, past medical and medication history, laboratory findings, surgery-related parameters including the type of surgery, type of general anesthetics used during the surgery, surgery and anesthesia time, the amount of intraoperative colloid administration and the incidence of transfusion [1,24,25]. The choice of general anesthetic agent was made by the attending anesthesiologists and there were no institutional guidelines followed for any type of surgery. The decision was made by the attending anesthesiologist based on patients’ comorbidity or baseline medical status. However, as there were significant variations in the choice of the main anesthetic agents, for the same type of surgery, according to many attending anesthesiologists over the long study period, we could hardly say that there was a consistent choice of anesthetic agent on the basis of patients’ comorbidity or baseline medical status. The maintenance of aspirin or clopidogrel was defined as maintenance until surgery without discontinuation during the seven days before surgery. Patients who discontinued aspirin or clopidogrel for 1 to 6 days were not included in our analysis because of the potential for a variable residual effect. Maintenance or discontinuation of aspirin or antiplatelet agent was determined after consultation by a cardiologist. Dual antiplatelet therapy or at least aspirin was maintained when the surgery was performed during the first 4 or 6 weeks following drug-eluting stent implantation, and the decision was made individually for other cases after taking into consideration the risk of thrombotic complications and excessive bleeding during surgery [26,27]. 

According to the anesthetic agent used during the surgery, the cohort was divided into three groups: TIVA, sevoflurane, and desflurane groups. In patients who received TIVA, a target-controlled infusion of propofol was performed via an infusion pump (Orchestra^®^; Fresenius Vial, Brezins, France). In patients who received sevoflurane or desflurane, anesthesia was induced by a propofol bolus dose of 1–2 mg/kg and maintained by either sevoflurane (2%–4 volume%) or desflurane (6%–8 volume%). The doses of anesthetic agents were adjusted with the guidance of the bispectral index. For balanced anesthesia, remifentanil was continuously infused using a target-controlled infusion in all patients.

### 2.3. Study Outcomes

The primary outcome of our study was the incidence of MACCE, which was defined as a composite of fatal or non-fatal myocardial infarction, pulmonary embolism, non-hemorrhagic stroke, or coronary revascularization within postoperative 30 days. A detailed definition of MACCE is given in Table 2. For the patients discharged within postoperative 30 days, the primary outcome was assessed from the follow-up medical record of our outpatient clinic. 

A major bleeding event was defined as follows [29]: (1) transfusion of 2 units or more of packed red blood cells during the surgery with a baseline hematocrit ≤30% or a drop of 10% or more from the baseline, or (2) requiring transfusion of ≥4 units of red blood cells within a 24-hour period, or (3) requiring any one of the following interventions: nasal packing, superficial vascular repair, arterial embolization, postoperative retroperitoneal, intraocular or intraspinal bleeding, or re-operation for surgical bleeding.

### 2.4. Statistical Analysis

Data were presented as mean (standard deviation) or median (interquartile range) or number (%). Normality of data distribution was determined by the Kolmogorov–Smirnov test. Continuous variables were compared between two groups by the Student *t*-test or the Mann–Whiney *U* test. A one-way analysis of variance or the Kruskal–Wallis test were used to compare continuous variables among the three groups. For categorical variables, the chi-square test or Fisher’s exact test were performed according to the expected counts. When we compared our study outcomes between any pair among the three groups before and after matching, *p*-values were adjusted by the Bonferroni correction to minimize the possibility of a type 1 error. Therefore, a *p*-value less than 0.008 (0.05/6) was indicated as statistically significant because we intended to control for the comparison of the three pairs of groups before and after matching. All statistical analyses were performed using the SPSS software (version 25.0; IBM Corp., Armonk, NY, USA) and STATA/MP (version 15.1; StataCorp, College Station, TX, USA). The values of less than 3% of the variables of baseline characteristics were missing. There was no missing date regarding study outcomes. In the case of continuous variables, the missing values were handled by single imputations using age- and sex-specific median values. Incidence data were replaced by the most frequently observed age- and sex-specific values. 

Firstly, to reduce the selection bias caused by the confounding factors before comparing the primary outcome between groups, four different pairwise propensity score matching analyses were performed. The matching was performed to adjust the baseline differences for the following pairs: TIVA vs. volatile anesthetics, TIVA vs. sevoflurane, TIVA vs. desflurane, and sevoflurane vs. desflurane. Propensity score matching considered the following covariates: age, sex, body-mass index, history of hypertension, diabetes mellitus, chronic kidney disease, and stroke, type of coronary stent, operation time, preoperative hemoglobin and serum albumin levels, type of surgery (emergency, vascular and musculoskeletal surgery), intraoperative colloid administration and red cell transfusion, maintenance of aspirin or clopidogrel, and time interval between coronary stent insertion and surgery. Matching was performed using the nearest neighbor algorithm with a caliper width of 0.1. We compared standardized mean differences to assess balance before and after matching. A standardized mean difference > 0.1 was considered imbalanced. The incidence of MACCE was compared between the matched groups.

Secondly, as a sensitivity analysis, multivariable logistic regression analysis for MACCE was performed in the total cohort to evaluate whether volatile anesthetics or total intravenous anesthesia is significantly associated with MACCE. No univariable screening was performed before multivariable analysis and no variable selection process was performed during the multivariable analysis.

Although the sample size was not determined prior to the analysis, the study power for the primary endpoint was calculated with the available number of patients for our study. With 975 and 655 patients available in TIVA and volatile anesthetics groups to compare the incidence of MACCE and the observed incidence of MACCE of these groups in our study, the study power was 71.3% to detect the observed difference. For pulmonary embolism, the study power was 80.9%. 

## 3. Results

A total of 1987 patients who underwent non-cardiac surgery after coronary stent implantation were initially identified. Among these, 209 patients were excluded due to the operation time being less than one hour and 148 were excluded because of the lack of information regarding stent type. Among the 1630 patients included in the final analysis, 975 patients (59.8%) received TIVA, and 655 patients received volatile anesthetics (40.2% including sevoflurane 26.9% and desflurane 13.3%) during the surgery. 

**Figure 1:**
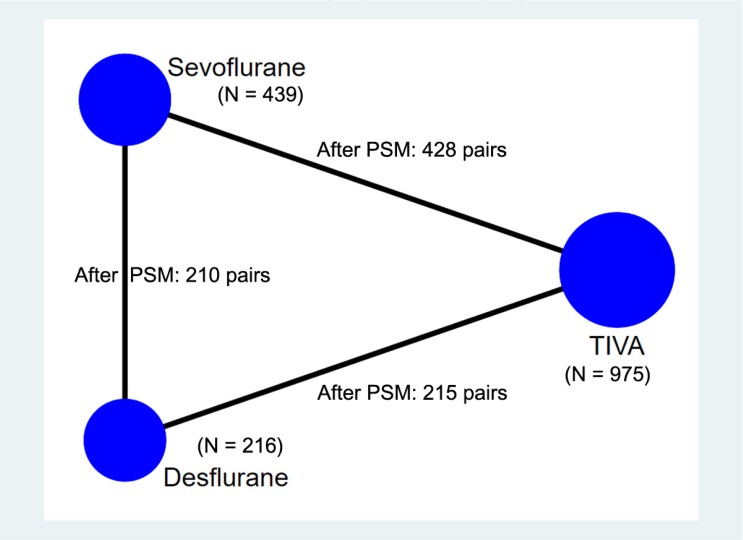
Network plot denoting the study group and number of patients in groups and propensity score matching. PSM = propensity score matching.

**Figure 2:**
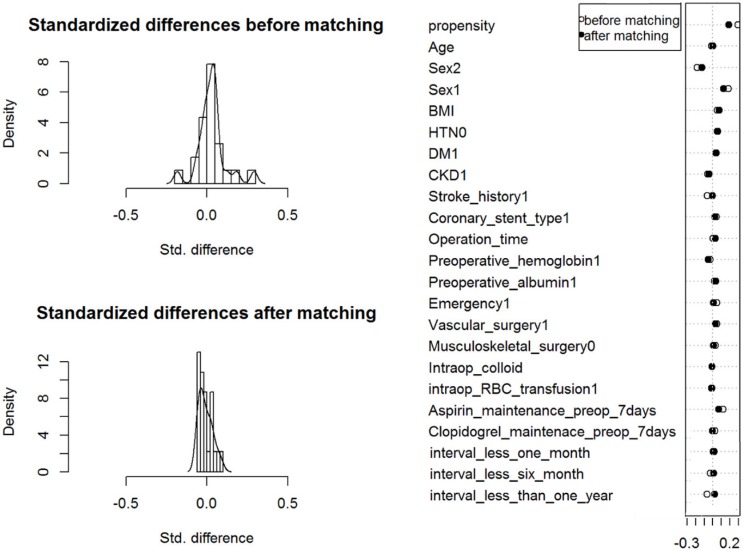
Histogram and covariate balance plot of distribution of propensity scores between patients with total intravenous anesthesia and volatile anesthetics. BMI = body-mass index; HTN = hypertension; DM = diabetes mellitus; CKD = chronic kidney disease; intraop = intraoperative; RBC = red blood cell; ASA = aspirin; interval = the interval between coronary stent insertion and surgery.

After matching, 642 pairs between TIVA and volatile anesthetics groups, 428 pairs between TIVA and sevoflurane groups, 215 pairs between TIVA and desflurane groups and 210 pairs between sevoflurane and desflurane groups remained (Figure 1). Histograms of the distribution of propensity scores and covariate balance plots before and after propensity score matching are presented in Figure 2 and Supplemental Figures S1–S3. There was no unbalanced contributor to the propensity scores with a standardized difference ≥ 0.20 between the groups after matching all pairs.

The baseline patient characteristics and surgery and anesthesia-related variables are summarized in Table 1. There were no cases involving bare-metal stents. The overall incidence of MACCE after noncardiac surgery within postoperative 30 days was 6.0% (fatal-myocardial infarction: *n* = 0, 0.0%; non-fatal myocardial infarction: *n* = 72, 4.4%; stroke: *n* = 14, 0.9%; pulmonary embolism: *n* = 11, 0.7%; and coronary revascularization: *n* = 8, 0.5%). Coronary revascularization was performed in patients with myocardial infarction. 

**Figure 3:**
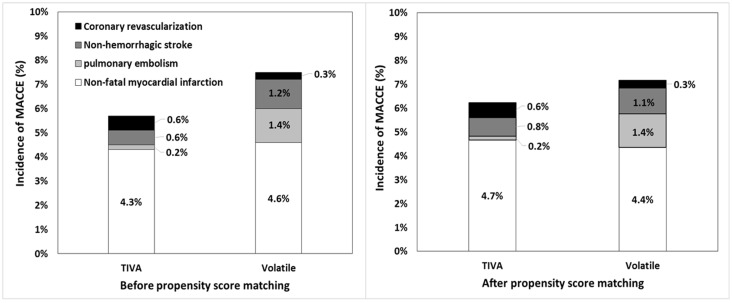
Frequency of postoperative major adverse cardiovascular and cerebral event in patients with total intravenous anesthesia, sevoflurane, or desflurane. SEVO = sevoflurane; DES = desflurane.

Before matching, the incidence of MACCE was significantly higher in the sevoflurane group than in the TIVA group (sevoflurane 8.2% vs. TIVA 5.1%, *p* = 0.006). However, there was no significant difference between the volatile and the TIVA groups (volatile group 7.2% vs. TIVA 5.1%, *p* = 0.087) (Figure 3). When the incidence of individual components of MACCE in respective groups was compared, the incidence of pulmonary embolism was significantly different between sevoflurane and TIVA groups (sevoflurane 1.6% vs. TIVA 0.2%, *p* = 0.005). However, after the propensity score matching was performed, the incidence of MACCE was not significantly different between the groups of any pair (TIVA 5.6% vs. volatile anesthetics 6.9%, *p* = 0.356; and TIVA 6.5% vs. sevoflurane 7.7%, *p* = 0.507) (Table 3 and Supplemental Tables S1–S3). The incidence of pulmonary embolism was also not significantly different between the groups of any pair (TIVA 0.0% vs. sevoflurane 1.6%; *p* = 0.015) (Table 3 and Supplemental Tables S1–S3).

The multivariable logistic regression analysis showed that the time interval of less than 30 days between coronary stent implantation and surgery, chronic kidney disease, surgery time, vascular surgery, and musculoskeletal surgery was significantly associated with the development of MACCE (Table 4). The maintenance of dual antiplatelet therapy was associated with a decreased risk of MACCE (OR 0.83, 95% CI 0.71–0.98, *p* = 0.041). The choice of volatile agents vs. TIVA was not significantly associated with MACCE (OR 1.49, 95% CI 0.91–2.39, *p* = 0.213).

## 4. Discussion

To the best of our knowledge, this is the first study investigating the association of intraoperative anesthetics with clinical outcomes after noncardiac surgery in patients with previous coronary stent implantation. After performing propensity score matching to reduce the effect of potential confounders, we found no difference in the incidence of MACCE between the different anesthesia groups. The multivariable logistic regression analysis also supports the finding that there is no significant association between the choice of the anesthetic agent and MACCE. However, the limitations of the single-center retrospective analysis with insufficient power should be considered while interpreting our study results. 

Some previous laboratory studies have reported the cardioprotective effects of volatile anesthetics [11,12,13] and propofol [14,15]. The molecular mechanism for explaining the cardioprotective effects of volatile anesthetics involves the attenuation of the toxic effects of reactive oxygen species [30,31]. Protein kinase C is considered an important mediator of the signaling pathway [32]. Volatile anesthetics stimulate the activity of protein kinase C [33,34] and hinder the opening of mitochondrial permeability transition pore (PTP), which is associated with cell death [35]. Propofol also has a cardioprotective property, which is related to enhancing the antioxidant capacity of the tissue [14]. Another study reported the protective effects of propofol as being associated with the reduced opening of the mitochondrial PTP, leading to decreased oxidative stress [15]. 

However, to our knowledge, there has been no clinical study investigating the association between anesthetic agents and postoperative MACCE in patients with a history of coronary stent insertion undergoing noncardiac surgery. Nonetheless, there were recommendations regarding the choice of general anesthetics in patients with a high risk of myocardial injury. Given the cardioprotective property of volatile anesthetics, the 2007 ACC/AHA Guidelines of Perioperative Cardiovascular Care for Noncardiac Surgery recommended the use of volatile anesthetics to maintain general anesthesia for noncardiac surgery in hemodynamically stable patients with an increased risk of developing myocardial ischemia [36]. Moreover, a randomized trial demonstrated that sevoflurane reduced the release of cardiac troponin I significantly more than propofol did in patients with coronary artery disease undergoing vascular surgery [20]. However, considering the accumulating evidence indicating the lack of significant benefits of choosing volatile anesthetics over propofol during noncardiac surgery [16,21,37,38,39], the 2014 ACC/AHA guidelines thus revised the recommendations regarding the choice of anesthetics for noncardiac surgery [26]. Subsequent randomized trials comparing TIVA with sevoflurane also reported no difference in their primary outcomes related to myocardial injury [22,40]. 

In case of cardiac surgical patients with a high risk of postoperative myocardial injury, previous meta-analyses have reported reduced mortality after cardiac surgery when volatile anesthetics were used compared to TIVA [41,42]. In another recent meta-analysis including 68 randomized trials, general anesthesia with volatile anesthetics was found to be associated with reduced mortality and a lower incidence of postoperative pulmonary complications following cardiac surgery [43]. This meta-analysis also presented the results of noncardiac surgery but there was no significant difference observed between the anesthetic agents, which is consistent with our results [43]. Additionally, for cardiac surgery, a recent large international multicenter randomized trial reported no significant difference in the one-year mortality rate between volatile anesthetics and TIVA [23]. 

Our logistic regression analysis revealed that a time interval of less than one month between stent insertion and surgery, chronic kidney disease, long surgery time and vascular and musculoskeletal surgeries is associated with MACCE after noncardiac surgery in these patients. Most of these predictors for MACCE have been reported by previous studies [4,5]. The maintenance of aspirin or clopidogrel until surgery was not found to be significantly associated with MACCE in our results, which does not agree with the previous guidelines [26]. However, the protective role of an antiplatelet agent against the major adverse cardiovascular event was reported to be insignificant by a recent prospective observational study [44]. Another recent retrospective study of patients with second-generation DES reported that the incidence of major adverse cardiac events was not influenced by antiplatelet therapy [45]. However, dual antiplatelet therapy showed cardioprotective effects in this study. The patients who received dual antiplatelet therapy in our study were mostly those who underwent noncardiac surgery within 1 month after coronary stent implantation. Our results support the strategy that dual antiplatelet therapy should be maintained in these patients, which was mentioned in the previous guidelines [3,26,36].

The strength of the present study lies in the fact that we attempted to reduce the selection bias and confounding effects of the covariates by performing a series of propensity score analyses. Matching was performed pairwise like a network analysis with independent matching for three pairs of general anesthetics. The consistent results obtained from different pairs of network comparison strongly support our conclusion. 

Interpretation of this study needs to be done cautiously as there are significant limitations to be considered. Firstly, since this study is a single-center retrospective analysis, unknown or unmeasured biases could have affected our results. External validity is limited. Although we included previously known perioperative variables that could affect MACCE in our propensity score analysis, residual confounding due to the excluded or unknown covariates and their interaction may still exist [46]. Secondly, although most cases of myocardial infarction were considered to be stent thrombosis, there were a few ambiguous cases in which stent thrombosis had to be determined by a medical chart review. Therefore, the effect of the anesthetic agent on the incidence of true stent thrombosis cannot be elucidated by our study results. Thirdly, the guidelines to manage the patients undergoing noncardiac surgery after coronary stent implantation were changed during the period when our data were being collected [3,26,36]. Compared to 2007 ACC/AHA Guideline on Perioperative Cardiovascular Evaluation and Care for Non-cardiac Surgery [36], 2014 ACC/AHA guidelines recommended a shorter acceptable duration of time until non-cardiac surgery needed to complete dual antiplatelet therapy after DES implantation [47]. Compared to the prior 12 months recommended by the 2007 guideline, the 2014 ACC/AHA guideline recommended a shorter delay for elective non-cardiac surgery after DES implantation. According to this, an elective non-cardiac surgery could be considered after six months if the risk of further delay is greater than the risk of ischemia and stent thrombosis [26]. The 2016 ACC/AHA-focused update on the duration of dual antiplatelet therapy in patients with coronary artery disease recommended that an elective non-cardiac surgery may be considered even after three months if the risk of further delay of surgery is greater than the risks of stent thrombosis [3]. Although we conducted a multivariable analysis and propensity score matching, significant heterogeneity in the timing of surgery and discontinuation of antiplatelet drugs could still be potential confounding factors. 

## 5. Conclusions

In our propensity score-matched comparison of MACCE in patients with a history of coronary stent implantation undergoing noncardiac surgery, we did not find any significant association between the choice of general anesthetic agent and the development of MACCE. There was also no significant difference in the incidence of any individual component of MACCE between different anesthesia groups in the matched cohorts. Given the single-center retrospective design, the insufficient power of our study, and the lack of previous studies regarding this topic, further studies with sufficient power are required to confirm our findings and elucidate the causal relationship between general anesthetic agents and MACCE in these high-risk patients.

## Figures and Tables

**Table 1:** Baseline characteristics of patients between anesthetics used during the surgery.

Characteristics	TIVA (*n* = 975)	SEVO (*n* = 439)	DES (*n* = 216)	*p*-Value
**Demographic data**				
Age, years, median (IQR)	69 (61–76)	70 (61–77)	71 (62–78)	0.307
Female, *n*	322 (33.0)	174 (39.6)	82 (38.0)	0.040
Body-mass index, kg/m^2^	23.4 (21.7–24.9)	23.4 (21.0–24.9)	23.4 (21.3–25.7)	0.305
Body-mass index >30 kg/m^2^, *n*	33 (3.4)	12 (2.7)	8 (3.7)	0.950
**Medical history**				
Hypertension, *n*	590 (60.5)	286 (65.1)	128 (59.3)	0.190
Diabetes mellitus, *n*	382 (39.2)	173 (39.4)	70 (32.4)	0.156
Stroke, *n*	21 (2.2)	16 (3.6)	4 (1.9)	0.203
Chronic kidney disease*, *n*	106 (10.9)	62 (14.1)	21 (9.7)	0.137
ASA physical status classification, 2/3/4 *n*	620 (63.6)/334 (34.3)/21 (2.2)	279 (63.6)/151 (34.4)/9 (2.1)	138 (63.9)/73 (33.8)/5 (2.3)	0.970
**Coronary intervention type**				
First generation drug-eluting stent, *n*	394 (40.4)	164 (37.4)	88 (40.7)	0.520
Sirolimus-eluting stent (Cypher), *n*	181 (18.6)	78 (17.8)	39 (18.1)	0.934
Paclitaxel-eluting stent (Taxus), *n*	222 (22.8)	87 (19.8)	54 (25.0)	0.273
Second generation drug-eluting stent (Xience, Endeavor, Resolute, Coroflex), *n*	581 (59.6)	275 (62.6)	129 (59.7)	0.540
Time from PCI to surgery, days	548 (132–1438)	647 (150–1456)	638 (144–1461)	0.595
Time from PCI to surgery				
<30 days	140 (14.4)	62 (14.1)	35 (16.2)	0.753
30–180 days	133 (13.6)	55 (12.5)	23 (10.6)	0.473
181–365 days	107 (11.0)	38 (8.7)	22 (10.2)	0.413
>1 year	595 (61.0)	284 (64.7)	136 (63.0)	0.410
Maintenance of aspirin until surgery without discontinuation, *n*	299 (30.7)	107 (24.4)	57 (26.4)	0.041
Maintenance of clopidogrel until surgery without discontinuation, *n*	139 (14.3)	53 (12.1)	34 (15.7)	0.379
Maintenance of dual antiplatelet agent, *n*	101 (10.4)	35 (8.0)	21 (9.7)	0.420
**Preoperative other medications**				
Beta-blocker, *n*	185 (19.0)	82 (18.7)	44 (20.4)	0.737
ACE inhibitor, *n*	65 (6.7)	31 (7.1)	15 (6.9)	0.817
Angiotensin receptor blocker, *n*	147 (15.1)	61 (13.9)	38 (17.6)	0.592
Calcium channel blocker, *n*	248 (25.4)	109 (24.8)	57 (26.4)	0.888
Statin, *n*	273 (28.0)	121 (27.6)	59 (27.3)	0.813
Diuretics, *n*	78 (8.0)	35 (8.0)	19 (8.8)	0.755
Oral hypoglycemic agent, *n*	361 (37.0)	163 (37.1)	65 (30.1)	0.118
**Preoperative laboratory finding**				
Hematocrit, %	38.3 (34.8–42.1)	38.2 (33.9–42.1)	39.4 (35.3–41.6)	0.303
Albumin, g/dL	4.1 (3.8–4.4)/*n* = 937	4.2 (3.9–4.4)/*n* = 428	4.2 (3.9–4.4)/*n* = 213	0.248
**Surgery-related parameter**				
High-risk surgery				
Emergency surgery, *n*	27 (2.8)	13 (3.0)	0 (0.0)	0.043
Vascular surgery, *n*	99 (10.2)	36 (8.2)	12 (5.6)	0.080
Intermediate-risk surgery				
Nose, mouth, and pharynx surgery, *n*	96 (9.8)	51 (11.6)	5 (2.3)	<0.001
Abdominal surgery, *n*	392 (40.2)	119 (27.1)	61 (28.2)	<0.001
Musculoskeletal surgery, *n*	128 (13.1)	78 (17.8)	15 (6.9)	0.001
Neurosurgery, *n*	27 (2.8)	20 (4.6)	8 (3.7)	0.218
Low-risk surgery				
Urologic surgery, *n*	112 (11.5)	35 (8.0)	56 (25.9)	<0.001
Gynecologic surgery, *n*	16 (1.6)	13 (3.0)	8 (3.7)	0.096
Miscellaneous, *n*	105 (10.8)	87 (19.8)	51 (23.6)	<0.001
Surgery time, min	130 (65–200)/*n* = 942	120 (65–209)/*n* = 422	120 (60–183)/*n* = 212	0.152
Anesthesia time, min	170 (100–255)/*n* = 941	170 (100–265)/*n* = 422	155 (95–235)/*n* = 212	0.056
Intraoperative colloid administration, *n*	171 (17.5)	83 (18.9)	22 (10.2)	0.015
Intraoperative colloid administration, mL	500 (500–900)	500 (500–1000)	500 (500–900)	0.240
Intraoperative red blood cell transfusion, *n*	66 (6.8)	35 (8.0)	11 (5.1)	0.384
Intraoperative fresh frozen plasma transfusion, *n*	57 (5.8)	29 (6.6)	10 (4.6)	0.598

Data are presented as number (%) or mean (SD) or median (interquartile range). For continuous variable, the number of patients was shown if there was missing. TIVA, total intravenous anesthesia; SEVO, sevoflurane; DES, desflurane; IQR, interquartile range; ASA, American Society of Anesthesiologists; PCI, percutaneous coronary intervention; ACE, angiotensin converting enzyme. * Chronic kidney disease was defined by at least two consecutive glomerular filtration ratio values <60 mL/min/1.73 m^2^ separated by an interval of at least three months or dependence on regular hemodialysis. The risk classification of surgery-related parameters was according to 2014 European Society of Cardiology/European Society of Anaesthesiology guidelines on non-cardiac surgery [27] and a previous risk prediction model by Glance et al. [28].

**Table 2:** Definitions of the components of the primary endpoint of major adverse cardiovascular and cerebral event.

Outcome	Definition
Myocardial infarction	Diagnosis of myocardial infarction required any one of the followings: 1. A typical rise of troponin or a typical fall of an elevated troponin detected at its peak post-surgery in a patient without a documented other explanation for the troponin change (e.g., pulmonary embolism) OR a rapid rise and fall of creatinine kinase-myocardial band (CK-MB). This finding should accompany one of the following: a. ischemic sign or symptom b. development of pathologic Q wave c. ECG changes indicative of ischemia d. coronary intervention (i.e., PCI or CABG surgery) e. new or presumed new cardiac wall motion abnormality on echocardiography or new or presumed new fixed defect on radionuclide myocardial imaging 2. Development of new pathological Q wave on an ECG if troponin levels were not obtained or were obtained at times that could have missed the clinical event.
Non-fatal myocardial infarction	Non-fatal myocardial infarction was defined as successful patient treatment and resuscitation from either documented or presumed myocardial infarction.
Coronary revascularization	Cardiac revascularization procedure was defined as PCI or CABG surgery.
Pulmonary embolism	The diagnosis of pulmonary embolism required any one of the following: 1. Diagnosis suggested with a high probability by ventilation/perfusion lung scan 2. An intraluminal filling defect on pulmonary angiography 3. An intraluminal filling defect of segmental or larger pulmonary artery on a helical CT scan 4. A positive diagnostic test for deep vein thrombosis and one of the following a. non-diagnostic ventilation/perfusion lung scan (i.e., low or intermediate probability suggested) b. non-diagnostic helical CT scan (i.e., subsegmental defect or technically inadequate study)
Non-hemorrhagic stroke	Stroke was defined as a new focal neurological deficit thought to be vascular in origin with signs or symptoms lasting more than 24 hours. Non-hemorrhagic stroke was identified by lack of hemorrhage in the brain imaging study including CT or MRI.

ECG, electrocardiography; PCI, percutaneous coronary intervention; CABG, coronary artery bypass graft; CT, computed tomography, MRI, magnetic resonance imaging.

**Table 3:** Comparisons of major adverse cardiovascular and cerebral event and major bleeding between patients with total intravenous anesthesia and volatile anesthetics before and after propensity score matching.

	Before Matching			After Matching		
Characteristics	TIVA (*n* = 975)	Volatile (*n* = 655)	*p*-Value	TIVA (*n* = 642)	Volatile (*n* = 642)	*p*-Value
MACCE	50 (5.1)	47 (7.2)	0.087	36 (5.6)	44 (6.9)	0.356
Fatal myocardial infarction	-	-	-	-	-	-
Non-fatal myocardial infarction	42 (4.3)	30 (4.6)	0.793	30 (4.7)	28 (4.4)	0.788
Pulmonary embolism	2 (0.2)	9 (1.4)	0.009	1 (0.2)	9 (1.4)	0.021
Non-hemorrhagic stroke	6 (0.6)	8 (1.2)	0.273	5 (0.8)	7 (1.1)	0.733
Coronary revascularization	6 (0.6)	2 (0.3)	0.487	4 (0.6)	2 (0.3)	0.687
Major bleeding	35 (3.6)	30 (4.6)	0.316	22 (3.4)	28 (4.4)	0.387

Data are presented as number (%). TIVA, total intravenous anesthesia; MACCE, major adverse cardiovascular and cerebral event.

**Table 4:** Multivariable logistic regression analysis to predict postoperative major adverse cardiovascular and cerebral event in patients undergoing noncardiac surgery after coronary stent implantation.

Variable	Odds Ratio (95% CI)	*p*-Value
Age, year	1.00 (0.98–1.03)	0.708
Female	0.70 (0.42–1.17)	0.174
Body-mass index > 30 kg/m^2^	0.68 (0.16–2.98)	0.612
Interval between PCI and surgery		
<30 days	2.10 (1.18–3.72)	0.011
30–180 days	1.02 (0.49–2.13)	0.963
181–365 days	0.86 (0.40–1.86)	0.697
>1 year	reference	
Hypertension	0.86 (0.54–1.39)	0.545
Diabetes mellitus	1.20 (0.77–1.88)	0.428
Chronic kidney disease	2.46 (1.39–4.35)	0.002
Stroke	0.86 (0.19–3.99)	0.847
Preoperative beta-blocker	0.89 (0.67–1.94)	0.435
Preoperative ACE inhibitor or ARB	0.95 (0.38–2.48)	0.514
Calcium channel blocker	1.35 (0.57–1.95)	0.774
Statin	0.91 (0.44–1.84)	0.614
Diuretics	1.13 (0.34–2.57)	0.546
Oral hypoglycemic agent	1.23 (0.60–1.74)	0.517
Second vs. first generation drug-eluting stent	0.97 (0.61–1.56)	0.906
Surgery time, hour	1.14 (1.03–1.26)	0.013
Preoperative hemoglobin, g/dL	0.91 (0.56–1.64)	0.400
Preoperative albumin, g/dL	0.92 (0.41–1.83)	0.205
Emergency surgery	1.39 (0.44–4.37)	0.575
Vascular surgery	2.84 (1.44–5.60)	0.003
Musculoskeletal surgery	2.59 (1.34–5.34)	0.002
Intraoperative colloid administration	1.00 (1.00–1.00)	0.327
Intraoperative red blood cell transfusion	1.41 (0.58–3.46)	0.448
Maintenance of aspirin until surgery without discontinuation	0.74 (0.36–1.39)	0.415
Maintenance of clopidogrel until surgery without discontinuation	0.52 (0.23–1.30)	0.245
Maintenance of dual antiplatelet therapy	0.83 (0.71–0.98)	0.041
Volatile anesthetics vs. total intravenous anesthesia	1.49 (0.91–2.39)	0.213

Data are presented as median (interquartile range) or number (%). CI, confidence interval; PCI, percutaneous coronary intervention; ACE, angiotensin converting enzyme, ARB, angiotensin receptor blocker.

## Supplementary Materials

The following are available online at https://www.mdpi.com/2077-0383/9/2/429/s1, Figure S1. Histogram and covariate balance plot of distribution of propensity scores between patients with total intravenous anesthesia and sevoflurane; Figure S2. Histogram and covariate balance plot of distribution of propensity scores between patients with total intravenous anesthesia and desflurane; Figure S3. Histogram and covariate balance plot of distribution of propensity scores between patients with sevoflurane and desflurane; Table S1. Comparisons of major adverse cardiovascular and cerebral event and major bleeding between patients with total intravenous anesthesia and sevoflurane before and after propensity score matching; Table S2. Comparisons of major adverse cardiovascular and cerebral event and major bleeding between patients with total intravenous anesthesia and desflurane before and after propensity score matching; Table S3. Comparisons of major adverse cardiovascular and cerebral event and major bleeding between patients with sevoflurane and desflurane before and after propensity score matching.
