# Supplementary material for: Anesthetic Agents and Cardiovascular Outcomes of Noncardiac Surgery after Coronary Stent Insertion
Source: J Clin Med. 2020 Feb 5;9(2):429. doi: 10.3390/jcm9020429 (PMC7074305; doi:10.3390/jcm9020429)
Supplement: Supplementary file 1 [file jcm-09-00429-s001.pdf]

Anesthetic Agents and Major Adverse Cardiovascular and Cerebral Event after Noncardiac Surgery in Patients with Coronary Stent Implantation

# : Supplemental Materials

Number	Title	Page
**Supplemental Figure S1.**	Histogram and covariate balance plot of distribution of propensity scores between patients with total intravenous anesthesia and sevoflurane.	2
**Supplemental Figure S2.**	Histogram and covariate balance plot of distribution of propensity scores between patients with total intravenous anesthesia and desflurane.	3
**Supplemental Figure S3.**	Histogram and covariate balance plot of distribution of propensity scores between patients with sevoflurane and desflurane.	4
**Supplemental Table S1.**	Comparisons of major adverse cardiovascular and cerebral event and major bleeding between patients with total intravenous anesthesia and sevoflurane before and after propensity score matching.	5
**Supplemental Table S2.**	Comparisons of major adverse cardiovascular and cerebral event and major bleeding between patients with total intravenous anesthesia and desflurane before and after propensity score matching.	6
**Supplemental Table S3.**	Comparisons of major adverse cardiovascular and cerebral event and major bleeding between patients with sevoflurane and desflurane before and after propensity score matching.	7

Supplemental Figure S1. Histogram and covariate balance plot of distribution of propensity scores between patients with total intravenous anesthesia and sevoflurane.

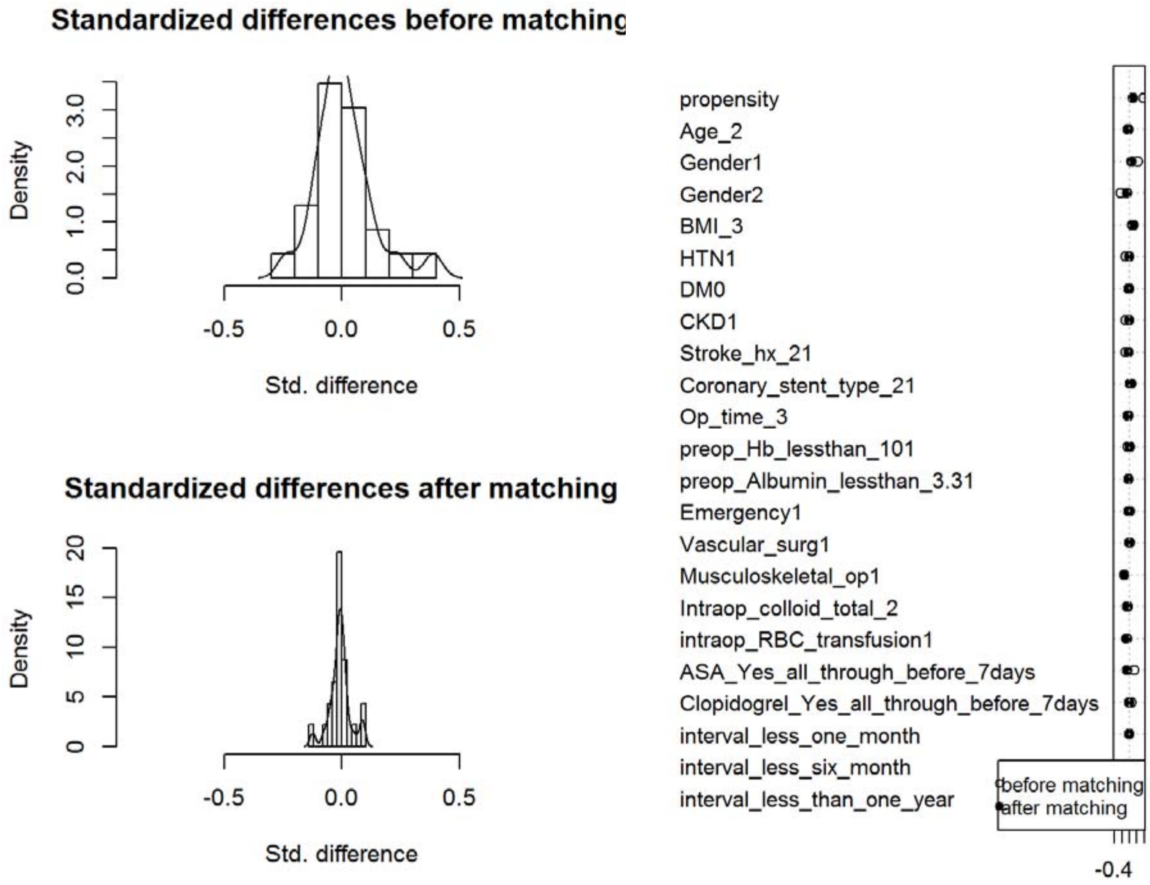

Supplemental Figure S2. Histogram and covariate balance plot of distribution of propensity scores between patients with total intravenous anesthesia and desflurane.

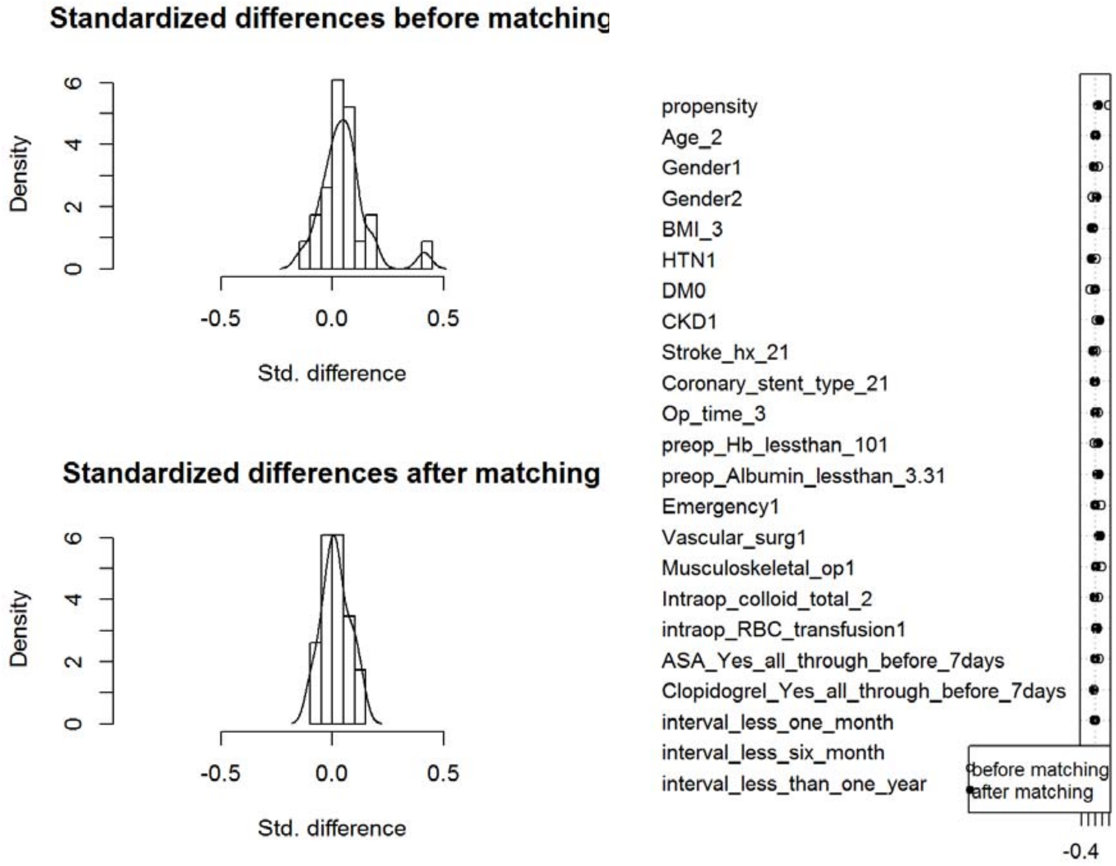

Supplemental Figure S3. Histogram and covariate balance plot of distribution of propensity scores between patients with sevoflurane and desflurane.

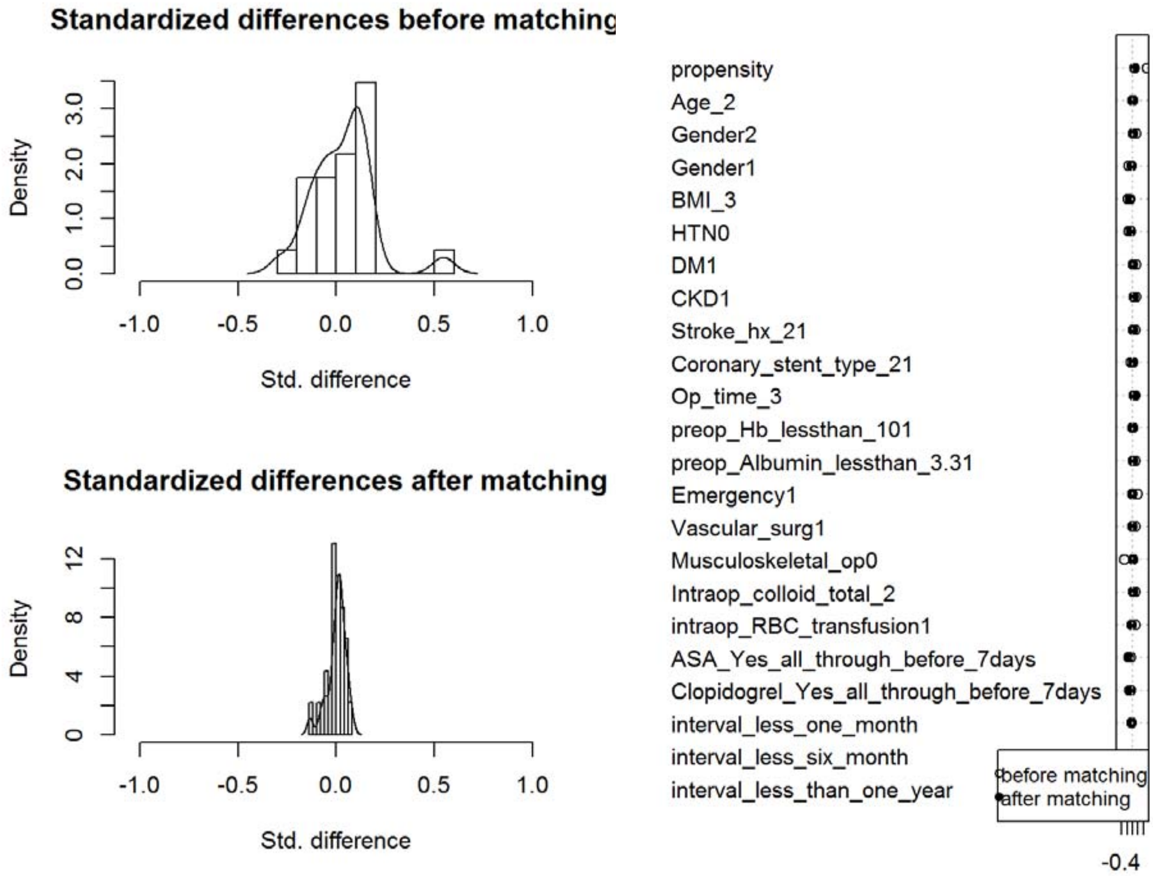

Supplemental Table S1. Comparisons of major adverse cardiovascular and cerebral event and major bleeding between patients with total intravenous anesthesia and sevoflurane before and after propensity score matching.

	Before matching			After matching		
Characteristics	TIVA (n=975)	SEVO (n=439)	*P*-value	TIVA (n=428)	SEVO (n=428)	*P*-value
MACCE	50 (5.1)	36 (8.2)	0.006	28 (6.5)	33 (7.7)	0.507
Fatal myocardial infarction	-	-	-	-	-	-
Non-fatal myocardial infarction	42 (4.3)	22 (5.0)	0.556	24 (5.6)	20 (4.7)	0.536
Pulmonary embolism	2 (0.2)	7 (1.6)	0.005	0 (0.0)	7 (1.6)	0.015
Non-hemorrhagic stroke	6 (0.6)	7 (1.6)	0.127	4 (0.9)	6 (1.4)	0.752
Coronary revascularization	6 (0.6)	1 (0.2)	0.447	3 (0.7)	1 (0.2)	0.624
Major bleeding	35 (3.6)	24 (5.5)	0.102	13 (3.0)	23 (5.4)	0.125

Data are presented as number (%).

TIVA, total intravenous anesthesia; SEVO, sevoflurane; MACCE, major adverse cardiovascular and cerebral event.

Supplemental Table S2. Comparisons of major adverse cardiovascular and cerebral event and major bleeding between patients with total intravenous anesthesia and desflurane before and after propensity score matching.

	Before matching			After matching		
Characteristics	TIVA (n=975)	DES (n=216)	*P*-value	TIVA (n=215)	DES (n=215)	*P*-value
MACCE	50 (5.1)	11 (5.1)	0.983	13 (6.0)	11 (5.1)	0.674
Fatal myocardial infarction	-	-	-	-	-	-
Non-fatal myocardial infarction	42 (4.3)	8 (3.7)	0.851	10 (4.7)	8 (3.7)	0.811
Pulmonary embolism	2 (0.2)	2 (0.9)	0.153	2 (0.9)	2 (0.9)	0.999
Non-hemorrhagic stroke	6 (0.6)	1 (0.5)	0.999	1 (0.5)	1 (0.5)	0.999
Coronary revascularization	6 (0.6)	1 (0.5)	0.999	1 (0.5)	1 (0.5)	0.999
Major bleeding	35 (3.6)	6 (2.8)	0.682	6 (2.8)	6 (2.8)	0.999

Data are presented as number (%).

TIVA, total intravenous anesthesia; DES, desflurane; MACCE, major adverse cardiovascular and cerebral event.

Supplemental Table S3. Comparisons of major adverse cardiovascular and cerebral event and major bleeding between patients with sevoflurane and desflurane before and after propensity score matching.

	Before matching			After matching		
Characteristics	SEVO (n=439)	DES (n=216)	*P*-value	SEVO (n=210)	DES (n=210)	*P*-value
MACCE	36 (8.2)	11 (5.1)	0.147	10 (4.8)	11 (5.2)	0.823
Fatal myocardial infarction	-	-	-	-	-	-
Non-fatal myocardial infarction	22 (5.0)	8 (3.7)	0.553	7 (3.3)	8 (3.8)	0.999
Pulmonary embolism	7 (1.6)	2 (0.9)	0.725	1 (0.5)	2 (1.0)	0.999
Non-hemorrhagic stroke	7 (1.6)	1 (0.5)	0.282	2 (1.0)	1 (0.5)	0.999
Coronary revascularization	1 (0.2)	1 (0.5)	0.551	1 (0.5)	1 (0.5)	0.999
Major bleeding	24 (5.5)	6 (2.8)	0.163	5 (2.4)	6 (2.9)	0.999

Data are presented as number (%).

SEVO, sevoflurane; DES, desflurane; MACCE, major adverse cardiovascular and cerebral event.
